# Second-Line Systemic Therapies in Metastatic Renal Cell Carcinoma: Current Insights and Future Directions

**DOI:** 10.33696/cancerimmunol.7.107

**Published:** 2025

**Authors:** Isaac E. Kim, Vivian Wong, Karie Runcie, Eric A. Singer

**Affiliations:** 1 Warren Alpert Medical School, Brown University, Providence, Rhode Island, USA; 2 Division of Urologic Oncology, The Ohio State University Comprehensive Cancer Center, Columbus, Ohio, USA; 3 Division of Hematology and Oncology, Columbia University Irving Medical Center, 161 Fort Washington Avenue, New York, NY, 10032, USA

**Keywords:** Metastatic renal cell carcinoma, Second-line treatment, immuno-oncology, Tyrosine kinase inhibitors, mTOR inhibitors, Immune-checkpoint inhibitors, HIF-2α inhibitors

## Abstract

Over the past few decades, the incidence of renal cell carcinoma (RCC) has rapidly increased with a considerable portion of patients presenting with metastatic disease (mRCC) and subsequent poor prognosis. Survival drops even further for those whose diseases progress on first-line therapy including immune-checkpoint inhibitors (ICIs) and vascular endothelial growth factor receptor (VEGFR) tyrosine kinase inhibitors (TKIs). In this review, we highlight the main second-line systemic therapies including TKIs, mTOR inhibitors, ICIs, and HIF-2α inhibitors along with their mechanisms of action and supporting clinical trials. We also highlight ongoing trials investigating novel second-line therapies such as the LITESPARK-011 trial contrasting belzutifan/lenvatinib with cabozantinib and the ENTRATA study examining glutaminase inhibitors including telaglenastat. The recent wave of key clinical trials has substantially increased the therapeutic options available to patients whose diseases have progressed on ICIs or VEGFR-TKIs. However, survival outcomes and the quality of life of mRCC patients on second-line treatments are still relatively limited, indicating a need for continued innovation and drug development in the field and continued trial recruitment at high-volume cancer centers.

## Introduction

Renal cell carcinoma (RCC) comprises 2% of all cancer diagnoses and deaths worldwide with an estimated annual incidence of 400,000 and 150,000 deaths [1,2]. Since 1975, the incidence of renal cell carcinoma has more than doubled in the U.S., resulting in an incidence of 82,000 and 15,000 deaths every year [3,4]. Two-thirds of these cases are localized, which has a 5-year survival rate of over 90% [5,6]. This survival rate, however, drops to 14% when the disease is metastatic (mRCC) [5,6].

For metastatic disease, first-line treatment typically consists of combination therapies with two immune-checkpoint inhibitors (ICIs) including anti-cytotoxic T-lymphocyte-associated protein 4 (CTLA-4) such as ipilimumab and programmed death-1 (PD-1) inhibitors such as nivolumab or an ICI combined with a vascular endothelial growth factor receptor (VEGFR) tyrosine kinase inhibitor (TKI) such as cabozantinib [7-9]. Unfortunately, the majority of patients experience disease progression after first-line therapy due to a variety of factors including tumor heterogeneity and significant adverse effects of first-line treatments [10-14]. With the emergence of biomarkers such as the tumor suppressor gene von Hippel Lindau (VHL), downstream modulator hypoxia-inducible factor (HIF), and the T-cell co-inhibitory signal programmed death ligand 1, several landmark trials over the past decade have ushered in a new wave of second-line systemic therapies including tyrosine kinase inhibitors, mTOR inhibitors, immune-checkpoint inhibitors (ICIs), and HIF-2α inhibitors.

## Second-Line Systemic Therapies

For patients who have disease progression after first-line treatment including adjuvant immune checkpoint inhibitors (ICIs), there has been much headway in second-line therapy options. Various factors such as patient age, comorbidities, preferences, past regimens, and biological tumor behavior should be considered when deciding on second-line systemic therapy options [15].

### Tyrosine kinase inhibitors

Tyrosine kinase inhibitors typically treat mRCC by inhibiting the intracellular domain of the VEGFR [16]. The main tyrosine kinase inhibitors are cabozantinib, axitinib, sunitinib, tivozanib, and lenvatinib (Table 1).

#### Cabozantinib:

Cabozantinib is a multikinase inhibitor that primarily targets VEGFR, c-MET, and AXL as well as other tyrosine kinases including FLT3, RET, and KIT [17]. Studies have found that cabozantinib prolonged survival in mRCC patients as second-line therapy after various therapies including VEGFR tyrosine-kinase inhibitors and/or ICIs.

In 2016, the open-label, phase 3 METEOR trial reported that patients with advanced or metastatic clear-cell RCC (ccRCC) who received cabozantinib after prior treatment with at least one VEGFR TKI experienced a median overall survival (OS) benefit of 21.4 months (95% CI 18.7-not estimable (NE)) compared to just 16.5 months for those on everolimus (14.7-18.8) (hazard ratio (HR) 0.66, 95% confidence interval (CI) 0.53-0.83, p=0.00026) [18,19]. Additionally, patients in the cabozantinib arm had increased progression-free survival (PFS) (HR 0.51, 0.41-0.62, p<0.0001) with a significant increase in objective response rates (ORR) (cabozantinib 17% vs. everolimus 3%, p<0.0001). Both arms had similar rates of serious adverse events (AEs) (cabozantinib 39% vs. everolimus 40%). Although only about 5% of patients in each study arm received prior ICI in the METEOR study, additional studies have confirmed the efficacy of cabozantinib as second-line therapy, including patients who progressed after initial ICIs.

Sazuka and colleagues reported on the first retrospective study of a Japanese cohort, specifically the Japanese Urological Oncology Group (JUOG) database, which included a total of 254 patients including 118 patients who used cabozantinib after ICI combination therapy [20]. These 118 patients experienced an ORR of 32%, disease control rate (DCR) of 75%, and median PFS of 10.5 months, indicating that cabozantinib after ICI was reasonably effective and feasible in clinical practice. Poor efficacy of cabozantinib after ICI combination therapy was associated with first-line treatment discontinuation related to progressive disease and liver metastasis.

There are also retrospective studies utilizing large real-world data sets to determine whether various first-line therapies followed by cabozantinib or the addition of new drugs combined with cabozantinib improve survival. The retrospective CABOSEQ study compared the effects of second-line cabozantinib in patients who received either first-line ipilimumab-nivolumab (IPI-NIVO), a combination of immuno-oncology and VEGFi (IOVE), or pazopanib or sunitinib (PAZ/SUN) and found that patients on first-line IPI-NIVO had a median OS of 21.4 months relative to 15.7 for the IOVE group and 20.7 for the PAZ/SUN group [21]. However, there were no statistically significant survival differences between the two groups (p=0.28) although the small sample size and retrospective design of the study may have limited the statistical power.

The multicenter, open-label Phase II CaboPoint trial is the first trial to investigate second-line cabozantinib in patients whose locally advanced or metastatic clear cell RCC progressed on ICI-based therapy. Patients were divided into two cohorts: cohort A, those who received first-line nivolumab and ipilimumab and cohort B, those who received a checkpoint inhibitor (CPI) and VEGFR TKI [5]. The primary endpoints were PFS, OS, and safety. In the final results reported in 2024, they reported that their patients observed an ORR of 40.5% (29.6-52.1) in cohort A vs. 27.5% (14.6-43.9) in cohort B, DCR of 84.8 months (75.0-91.9) vs. 80.0 (64.4-90.9), and median PFS of 10.9 months (8.2-14.2) vs. 8.3 (5.6-11.1) according to an independent review committee (IRC) and median OS of 24.3 months (18.5-31.8) vs. 24.1 (17.1- Not calculable) by investigator review (IR) [22]. Common treatment-related AEs included hypertension and diarrhea, resulting in treatment interruption among a significant proportion of patients (cohort A: 88%, cohort B: 76%) and discontinuation in 19% of patients. 29% of cohort A patients and 17% of cohort B patients experienced serious treatment emergent adverse events (TEAEs). Ultimately, the authors concluded that cabozantinib was effective in mRCC patients after ICI combination therapy and reported no new safety signals.

In a retrospective study comparing cabozantinib to other TKIs such as axitinib, lenvatinib, pazopanib, sorafenib, and sunitinib, Marteau and colleagues found that patients on cabozantinib experienced a higher 6-month response rate of 50.8% vs. 33.3% (p<0.001), higher overall response rate of 53.5% vs. 38.3% (p=0.041), and twice as long time to treatment discontinuation (TTD) [23]. Additionally, in an analysis stratified by metastatic location, patients with liver metastases on a combination of cabozantinib and nivolumab experienced higher PFS and OS compared to those on sunitinib (PFS: HR 0.51, 95% CI 0.33-0.79; OS: HR 0.47, 95% CI 0.27-0.82) [24].

The single-arm, phase 2 BREAKPOINT trial investigated whether different ICI combinations as first-line therapies affect the efficacy of cabozantinib as a second-line drug [25]. They reported a median PFS (mPFS) of 8.3 months (90% CI 3.9-17.4), median OS (mOS) of 13.8 months (95% CI 7.7-29.0), ORR of 37.9%, and a manageable rate of grade 3-4 AEs at 47%. Subsequently, the BREAKPOINT trial successfully achieved its primary endpoint [25].

#### Sorafenib, axitinib, and tivozanib:

Sorafenib, axitinib, and tivozanib are tyrosine kinase inhibitors that decrease angiogenesis and tumor growth by targeting various tyrosine kinases. Sorafenib helps induce apoptosis by inhibiting the Raf/MEK/ERK cascade and various serine/threonine and tyrosine kinases such as RAF1, BRAF, VEGFR 1, 2, 3, PDGFR, KIT, FLT3, fibroblast growth factor receptor (FGFR) 1, and RET [26,27]. Axitinib primarily inhibits VEGFR-1, 2, and 3 [28]. Tivozanib also mostly targets VEGFR-1, 2, and 3, although at higher concentrations, it can target other kinases including c-KIT and PDGFR [29].

In 2007, a phase 3, randomized, placebo-controlled study, the TARGET trial, reported that patients on sorafenib who progressed after initial systemic therapy experienced an increased PFS of 5.5 months relative to 2.8 months in those receiving placebo (HR 0.44, 95% CI 0.35-0.55, p<0.01). At the time, cytokines were the standard of care first-line treatment despite limited success and significant toxicity, so all patients in this trial received nephrectomy and cytokines including IL-2 and interferon alfa. However, patients on sorafenib were more likely to experience serious adverse events such as hypertension and cardiac ischemia [30]. Since then, several studies have identified more effective second-line tyrosine kinase inhibitors such axitinib, making the use of sorafenib largely historical. The AXIS trial discovered that patients on axitinib had a median PFS of 6.7 months compared to 4.7 months for those on sorafenib (HR 0.665, 95% CI 0.544-0.812, p<0.0001) [31].

Compared to sorafenib, the TIVO-3 trial examined the use of tivozanib as third-line therapy for mRCC patients. Specifically, they determined that patients on tivozanib who progressed on at least two systemic therapies including a VEGFR inhibitor had a median PFS of 5.6 months (95% CI 5.29-7.33), while those on sorafenib had a median PFS of just 3.9 months (95% CI 3.71-5.55) (HR 0.73, 95% CI 0.56-0.94, p=0.016). Additionally, patients tolerated tivozanib slightly better than sorafenib with a serious treatment-related adverse event (TRAE) rate of 11% on tivozanib compared to 10% on sorafenib [32].

#### Sunitinib:

Sunitinib mainly demonstrates binding activity to VEGFR-1, 2, and 3, PDGFR-α, PDGFR-β, and fibroblast growth factor receptor 1 along with some additional activity to cKIT, fms-related tyrosine kinase 3 (FLT3), rearranged during transfection (RET), and colony stimulating factor 1 receptor [33]. Given the superiority of cabozantinb to sunitinib, sunitinib is not often used as a second-line treatment option [34]. Thus, other TKIs including cabozantinib, axitinib, and tivozanib are often used instead.

A phase II, single-arm multicenter study known as the IMMUNOSUN trial reported that 19% of patients experienced an objective and partial response (95% CI 2.3-35.8), while an additional 67% had a stable response and 85.7% experienced a clinical benefit (95% CI 70.7-100.0) [35]. The mPFS and mOS were 5.6 and 23.5 months, respectively (mPFS: 95% CI 3.1-8.0, mOS: 95% CI 6.3-40.7). While the IMMUNOSUN trial failed to reach the pre-specified endpoint of a 30% ORR, it did show that sunitinib could be safely used as second-line therapy among mRCC patients after ICI combination therapy. A retrospective cohort study by Wells *et al.* observed a similar ORR of 22.5% as well as a 15.6-month median OS (95% CI 9.8-21.7), 57% 1-year OS rate (95% CI 45.2-68.0), and median time-to-treatment discontinuation of 5.4 months (95% CI 4.2-7.2) in patients receiving second-line sunitinib after first-line ICI therapy [36,37]. These findings confirmed that sunitinib had clinical activity even after first-line ICI treatment.

#### Lenvatinib:

For patients whose metastatic disease progressed after VEGFR-targeted therapy, they may benefit from lenvatinib alone or in combination with everolimus. In addition to VEGFR-1, −2, and −3, lenvatinib targets several tyrosine kinase receptors including FGFR-1, −2, −3, −4, PDGFRa, RET, and c-KIT [38]. Motzer and colleagues identified a significant PFS benefit among patients on lenvatinib alone or combined with everolimus compared to those on everolimus [39]. Specifically, compared to those on everolimus with a median PFS of 5.5 months (95% CI 3.5-7.1), patients on lenvatinib and everolimus had a median PFS of 14.6 months (5.9-20.1) (HR 0.40, 95% CI 0.24-0.68, p=0.0005). Those on lenvatinib alone also experienced a survival benefit compared to patients only on everolimus (HR 0.61, 95% CI 0.38-0.98, p=0.048). However, grade 3 and 4 events including diarrhea and proteinuria did occur more often in patients in the lenvatinib (79%) or lenvatinib/everolimus arms (71%) compared to those on everolimus only (50%). Thus, in 2016, the U.S. Food and Drug Administration (FDA) approved everolimus combined with lenvatinib for mRCC patients who previously underwent antiangiogenic therapy [40].

### mTOR inhibitors

The mechanistic or mammalian target of rapamycin (mTOR) signaling pathway is involved in cancer cell proliferation and survival and is usually active in mRCC [41]. mTOR also induces tumor angiogenesis and modulates hypoxia-inducible factors [41]. Studies have reported that 28% of clear cell RCC patients have gain-of-function mutations of the PI3K/AKT/mTOR signaling pathway, resulting in decreased survival [42]. Patients with certain molecular markers such as alterations in the PI3K/AKT/MTOR pathway, which may put them at risk for more aggressive disease, as well as TSC1/2 expression changes, PTEN epigenetic suppression, and VHL gene inactivation may be most likely to benefit from mTOR therapies [45-47].

In 2012, the phase III RECORD-1 trial investigated the survival effect of the mTOR inhibitor everolimus on PFS in mRCC patients who had previously received VEGFR-TKI therapy. It found that patients on everolimus who received one prior VEGRr-TKI experienced an mPFS of 5.4 months compared to just 1.9 months for those on placebo (HR 0.32, 95% CI 0.24-0.43, p<0.001). The lack of a non-placebo arm is likely attributable to the limited availability of other drugs in the early TKI era. Patients with two prior VEGFR-TKIs in the everolimus arm had a median PFS of 4.0 months, while those in the placebo arm had a median PFS of 1.8 months (HR 0.32, 95% CI 0.19-0.54, p<0.001) [43]. As noted previously, studies have also found moderate success with combination therapies consisting of mTOR inhibitors such as lenvatinib and everolimus and telaglenastat and everolimus although there are concerns about TRAEs [39,44,45]. The combination of lenvatinib and everolimus may also benefit patients with metastatic non-ccRCC such as papillary and chromophobe [46].

In patients who underwent ICI instead, however, the data is less clear [47]. One retrospective study found that among patients on lenvatinib and everolimus, those who were previously treated with ICI had a median PFS of 6.4 months vs. 5.7 months for those treated with TKI, suggesting that this drug combination had a survival benefit irrespective of prior treatment [48].

### Immune-checkpoint inhibitors

ICIs act by targeting the surface receptors linked to immune tolerance in tumor or immune cells [49]. Tumor proliferation occurs when the cell’s normal checking systems are overridden [50]. In the case of RCC, malfunctioning of the checking systems often involves T-cell co-inhibitory signals such as programmed death protein 1 (PD-1) and programmed death ligand 1 (PD-L1) [51]. By blocking PD-(L)1, immune-checkpoint inhibitors such as pembrolizumab, atezolizumab, and nivolumab promote activation of T-cells targeting tumor cells [52] (Table 2).

#### Pembrolizumab:

While used in first-line and adjuvant settings, the humanized monoclonal IgG4 kappa anti-PD1 antibody pembrolizumab has also been investigated as part of second-line treatment after prior ICI therapy [53,54]. In a phase 1b/2 study, Lee and colleagues reported that patients with mRCC on pembrolizumab and lenvatinib who had two previous ICI treatment lines had an ORR of 55.8% (95% CI 45.7-65.5), the proportion of patients with either complete or partial response as evaluated by radiology scans [55]. 66% of patients had grade 3 or higher AEs, and the most common grade 3 AE was hypertension [55].

A recent 2024 retrospective study also examined the survival effects of first-line systemic therapy after adjuvant ICIs including pembrolizumab, atezolizumab, and nivolumab/ipilimumab in recurrent RCC [56]. With most patients receiving first-line (1L) VEGF-targeted therapy (VEGF-TT), ICI + VEGF-TT, or ICI + ICI, the study reported an 18-month PFS and OS rate of 45% (95% CI 34-60) and 85% (95% CI 75-95), respectively, along with a TRAE rate of 42% [56]. They ultimately concluded that adjuvant pembrolizumab increased OS in RCC [56].

#### Atezolizumab:

The PD-L1 inhibitor atezolizumab may be used in some mRCC patients although it is not the standard of care. The phase 3 IMmotion 151 trial reported that there were no OS differences among treatment-naïve patients who received atezolizumab/bevacizumab vs. sunitinib (median OS: AB 36.1 months vs. sunitinib 35.3 months) despite a modest PFS benefit in the PD-L1-positive group (median PFS: AB 11.2 months vs. sunitinib 7.7 months; HR 0.74, 95% CI 0.57-0.96, p=0.0217) [57,58]. The phase 2 IMmotion150 trial examined patients who received atezolizumab combined with bevacizumab (AB) in mRCC patients whose disease progressed on atezolizumab or sunitinib [59]. It found that patients who received this second-line combination of bevacizumab and atezolizumab had a reasonable ORR of 27% (95% CI 19-37) and median additional PFS of 8.7 months (5.6-13.7) along with a TRAE rate of 83% and grade 3-4 TRAE rate of 30% [59]. The CONTACT-03 trial, a multicenter phase 3 trial conducted across 15 countries in Asia, Europe, North America, and South America, also found no PFS benefit among mRCC patients with the addition of atezolizumab to cabozantinib (AC) (Table 2) [median PFS: AC 10.6 months (95% CI 9.8-12.3) vs. cabozantinib 10.8 months (10.0-12.5), HR 1.03, 95% CI 0.83-1.28, p=0.78); median OS: AC 25.7 months (95% CI 21.5-NE) vs. cabozantinib NE (21.1-NE), HR 0.94, 95% CI 0.70-1.27, p=0.69)] [60]. Moreover, 48% of patients on AC experienced serious AEs compared to just 33% of those on cabozantinib, indicating that the addition of atezolizumab to cabozantinib increased toxicity [60]. In 2024, a separate, retrospective study on a real-world US-based database confirmed the lack of survival benefit of PD-1/L1 inhibitors combined with cabozantinib among a propensity score-matched cohort (real-world time to next therapy: HR 0.74 [0.49-1.12], real-world overall survival: HR 1.15 [0.73-1.79]) [61].

#### Nivolumab:

Nivolumab is a human IgG4 PD-1-targeting monoclonal antibody [62]. The CheckMate 025 trial studied patients who previously received one or two antiangiogenic first-line treatments and a maximum of three prior systemic therapies including cytokines and cytotoxic chemotherapy drugs such as sunitinib, pazopanib, and axitinib. The study showed that mRCC patients on nivolumab as second-line therapy survived longer than those on everolimus [63]. Specifically, these patients had an ORR of 25% vs. 5% (odds ratio 5.98, 95% CI 3.68-9.72, p<0.001), median OS of 25.0 months (95% CI 21.8-NE) vs. 19.6 months (17.6-23.1) (HR 0.73, 98% CI 0.57-0.93, p=0.002), and mPFS of 4.6 months (95% CI 3.7-5.4) vs. 4.4 months (3.7-5.5) (HR 0.88, 0.75-1.03, p=0.11). Grade 3-4 TRAEs were lower in nivolumab patients (19%) vs. everolimus (37%).

#### Evidence against ICI rechallenge:

So far, two prospective studies have suggested that ICI as second-line therapy may not confer a survival benefit among patients who already received an initial ICI. The TiNivo-2 trial determined that post-ICI mRCC patients who received tivozanib-nivolumab had a median PFS of 5.7 months, while those on tivozanib monotherapy had a median PFS of 7.4 months (Table 2) [64]. Similarly, the CONTACT-03 trial reported that the addition of atezolizumab to cabozantinib not only failed to confer a survival benefit but also increased toxicity, suggesting that mRCC patients should not be considered for ICI rechallenge [60].

A few promising studies have shown, however, that predictive biomarkers may provide key insight into immune-resistance mechanisms associated with ICI rechallenge [65]. ICIs decrease vasculature, which induce secondary hypoxia. Eventually, tumor cells can adapt to this hypoxic environment and develop resistance to ICIs by upregulating the HIF pathway [66,67]. Thus, the LITESPARK-003 trial determined that ICIs combined with HIF-2α inhibitors such as belzutifan may help boost ICI rechallenge by simultaneously addressing several VHL-associated pathways. Specifically, the trial reported a 31% ORR (95% CI 19-45) and median PFS of 13.8 months (95% CI 9-19) in patients on prior immunotherapy who received cabozantinib and belzutifan [65,68,69]. Tumor cells may also upregulate immune checkpoint markers by increasing PD-L1 expression, ultimately leading to T-cell apoptosis and exhaustion [73-76].

### HIF-2α inhibitors

Mutations in the von Hippel-Lindau (*VHL*) gene stabilize the hypoxia-inducible factor (HIF) transcription factor, which prevents proteasomal degradation and stimulates angiogenesis and tumorigenesis [65,70-73]. As a result, 70% of Von Hippel-Lindau patients who have germline mutations in *VHL* have early-onset ccRCC, and 50% of sporadic ccRCC cases have somatic *VHL* mutations [70,74].

In 2021, the phase 2, single-arm MK-6482-004 trial reported that the HIF-2α inhibitor belzutifan had an ORR of 49% (95% CI 36-62) and showed activity in RCC and non-RCC tumors associated with VHL disease [75]. This finding led to the FDA approval of belzutifan for VHL-associated tumors in 2021 [76].

Several trials are underway on the efficacy of belzutifan as second-line therapy for mRCC. For instance, the phase 3 LITESPARK-011 trial is investigating the differences in PFS between belzutifan/lenvatinib and cabozantinib in mRCC patients who previously received anti-PD-1/PD-L1 therapy [77]. The phase 2 LITESPARK-003 study examines the effects of belzutifan and cabozantinib on treatment-naïve advanced ccRCC patients (cohort 1) as well as those who received prior immunotherapy and 1-2 systemic therapies (cohort 2). Preliminary results revealed a median duration of response (DOR), PFS, OS, and ORR of 28.6 months (range 1.9-35.8), 30.3 months (95% CI 16-not reached), NR (95% CI NR-NR), and 70% (95% CI 55-82) respectively, in cohort 1 and 31.5 months (4.2-36.8), 13.8 months (95% CI 9-19), 26.7 months (95% CI 20-41), and 79% (95% CI 59-92) in cohort 2 [68]. Similarly, the phase 1/2 adaptive umbrella KEYMAKER-U03B trial is currently investigating the use of belzutifan monotherapy as second-line treatment and combined with the VEGF-TKI lenvatinib in patients who underwent anti-PD-1/L1 treatment [78]. It recently published preliminary results on the 32 patients it has accrued thus far. Among patients who received at least 2 post-baseline scans, the ORR was 50% (95% CI 29-71) with all partial responses and a clinical benefit rate (CBR) of 54% (95% CI 33-74). 74% of the responders were still responsive after 12 months, and the DOR ranged from 1.4 to 14.0 months although a median DOR was not reached. In the overall cohort, the median PFS was 11.2 months (95% CI 4-NR) with a clinical benefit rate of 55%. Additionally, while 93% of patients reported TRAEs including anemia, fatigue, and hypertension along with 50% experiencing grade 3-4 TRAEs, none have died yet from TRAEs. Thus, this trial has generally demonstrated auspicious antineoplastic activity in patients who progressed on first-line anti-PD-1/L1 and VEGF-TKI therapy.

Finally, the ongoing LITESPARK-005 trial compares belzutifan with everolimus in advanced ccRCC patients who received 1-3 previous systemic treatments including anti-PD-(L)1 inhibitors and VEGF-TKIs [79,80]. It recently published its preliminary results, which found that 33.7% and 22.5% of patients were progression-free on belzutifan vs. 17.6% and 9.0% on everolimus. Time to deterioration was also longer in belzutifan (median not reached) compared to everolimus (12 months) (HR 0.53, 95% CI 0.41-0.69, nominal p<0.0001). Unlike other second-line therapies such as VEGF-TKIs and ICIs, belzutifan does not have cardiovascular or gastrointestinal adverse effects. Rather, patients on belzutifan may experience hypoxia and anemia due to the drug’s inhibitive effect on erythropoietin [81]. Overall, belzutifan demonstrated a promising safety profile, and as a result, in December 2023, the FDA approved belzutifan for mRCC patients who had previously received an anti-PD-(L)1 inhibitor and VEGF-TKI [82].

Ongoing trials are also investigating the effect of combination therapies including HIF-2α inhibitors. Notably, the phase 2 LITESPARK-003 trial analyzed the ORR of patients on belzutifan and cabozantinib in both treatment-naïve and immunotherapy patients. As mentioned prior, preliminary results on this treatment regimen were promising with an ORR of 31% among patients who received prior immunotherapy [68]. While the final analysis on the immunotherapy cohort has yet to be published, in January 2025, the authors auspiciously reported that 70% of treatment-naïve patients who received belzutifan and cabozantinib experienced a confirmed objective response [83].

## The Role of Precision Medicine: Genomics, Liquid Biopsies, and Biomarkers

Given RCC’s heterogeneous nature, it is likely that precision medicine ranging from genomics to liquid biopsies to predictive biomarkers will play an increasingly important role in the treatment of RCC [84]. Studies have reported that patients with PBRM1 or BAF180 tumor suppressor genes near VHL may better tolerate treatment with anti-VEGF therapies [85]. Additionally, the CheckMate 025 trial found that the PBRM1 mutation was associated with improved outcomes for patients on nivolumab [86]. Regarding the utility of liquid biopsies in RCC treatment, Feng and colleagues revealed that mRCC patients on sorafenib who experienced a partial response also had decreased levels of cell-free DNA in contrast to those with progressive disease who had increased levels [87,88].

Predictive biomarkers for mRCC include histological biomarkers such as pathologic stage and histologic variant as well as genomic biomarkers [84]. It has been reported that tumors with a higher proportion of clear cells tend to respond better to anti-VEGF therapy [84,89,90]. Patients with a functional somatic mutation in BAP1 on everolimus and sunitinib experienced worse outcomes compared to the patients with the wild-type counterparts [91,92]. Similarly, a truncating mutation of PBRM1 has shown promise as a predictive biomarker for patients on ICIs [93]. Evidently, these biomarkers could potentially inform second-line treatment selection. These biomarkers may also provide insight into tumor resistance patterns to therapies such as ICIs as described prior and VEGFR-TKIs. Via upregulation of VEGF proangiogenic factors including fibroblast growth factor receptors, tumor cells can develop resistance to VEGFR-TKIs [66,67,94,95]. Motzer and colleagues then showed that patients whose disease progressed on certain VEGFR-TKIs such as sunitinib could benefit from the use of everolimus combined with lenvatinib, which inhibits FGFRs [39]. Nevertheless, few biomarkers have actually been validated for this purpose in mRCC, which remains a critical barrier to their use in clinical practice [84].

## Future Directions

In the rapidly changing space of second-line systemic therapies, special attention should be paid to ongoing trials including the LITESPARK-011 trial comparing belzutifan/lenvatinib vs. cabozantinib. A current phase II trial is determining the effect of belzutifan/cabozantinib in mRCC patients treated with ICIs [80]. Additionally, studies have found overexpression of glutaminase in RCC cell lines, paving the way for investigational studies on the effect of glutaminase inhibitors in mRCC [96]. Specifically, telaglenastat is an oral, selective, and potent glutaminase inhibitor that impedes RCC cell survival [97-100]. The phase II ENTRATA study determined that among mRCC patients who received two prior therapies including at least one VEGFR-TKI, those who received telaglenastat and everolimus (TelaE) had a median PFS of 3.8 months compared to 1.9 for those on placebo and everolimus (PboE) (HR 0.64, 95% CI 0.34-1.20, p=0.079) [44]. 74% of TelaE patients experienced grade 3-4 treatment-emergent adverse events (TEAEs) compared to 61% of PboE patients. In 2022, however, the phase III CANTATA trial found no added benefit of telaglenastat with cabozantinib compared to placebo with cabozantinib as second or third-line therapy in mRCC or mRCC patients who received at least 1 prior VEGFR-TKI or nivolumab and ipilimumab [101]. Specifically, Tela/Cabo patients observed an mPFS of 9.2 months vs. 9.3 months for Pbo/Cabo patients, ORR of 31% vs. 28%, and grade 3-4 TEAE rate of 71% vs. 79%, putting the clinical benefit of telaglenastat into question [101].

There are also important ongoing trials investigating first-line systemic therapies. For example, the phase III LITESPARK-012 trial is also examining the survival benefit of pembrolizumab and lenvatinib with or without belzutifan or the CTLA-4 antibody quavonlimab as first-line therapies for metastatic clear cell RCC [102].

Clinical trials on multistage therapies should also be carefully designed to account for salvage therapies, as conventional approaches to estimate OS typically focus on the initial treatment only [103]. Consequently, during the International Kidney Cancer Symposium (IKCS) North America in 2023, a think tank published a proposal composed of multistage treatment strategies and Sequentially Multiple Assignment Randomized Trials (SMARTs) to address comparisons between multistage regimens [104]. Additionally, they called for increased patient engagement and the incorporation of information technology during the design of trials [104].

Finally, certain populations including patients with central nervous system (CNS), liver, and bone metastases, and aggressive histologic features as well as elderly, frail, and immunosuppressed patients are often underrepresented in clinical trials on second-line treatments for mRCC [105]. These populations often experience increased mortality rates. For instance, a recent study determined that while 8.1% of advanced RCC patients had brain metastases upon initiating systemic therapy, they observed a significantly shorter median OS (22.8 months, 95% CI 18.3-25.9 vs. 34.7 months, 95% CI 32.7-37.0; p < 0.001) [106]. While some trials have included these patients, there is a need for subgroup analyses, as these drugs may affect these populations differently. For instance, the METEOR and BREAKPOINT trials included patients with non-symptomatic brain metastases but did not perform subgroup analyses [18,19,25].

However, the few trials that have specifically examined second-line therapies in these underrepresented populations have had significant clinical implications. The GETUG-AFU 26 NIVOREN phase II trial discovered limited activity of nivolumab in metastatic ccRCC patients with untreated brain metastases compared to those who had their brain metastases treated with prior therapy [107]. This trial has significant implications on this population of patients, as it suggests that they should undergo focal therapy for their brain metastatic lesions before being treated with ICIs. Evidently, the few trials that have specifically examined second-line therapies in these underrepresented populations have had significant clinical implications. Finally, emerging treatments and new clinical trials should focus on patient-centric outcomes including quality of life, tolerability, patient preference, and patient satisfaction in addition to the typical survival metrics [108]. Li *et al.* proposed the adoption of patient-centric clinical trials where patient needs are prioritized across all stages of the trial from design and enrollment to data collection and analysis [109]. Considering these objectives is especially important in the treatment of mRCC where curative therapies may not be feasible [108].

## Conclusion

Thanks to the advent of several new clinical trials over the past few years, the field of second-line systemic therapies for mRCC has rapidly grown. Patients with mRCC whose diseases have progressed on ICIs or VEGFR-TKIs have many options available to them including tyrosine kinase inhibitors, mTOR inhibitors, glutaminase inhibitors, ICIs, and HIF-2α inhibitors although few have significantly improved survival. Specific treatment options are often selected based on factors such as patient age, comorbidities, first-line therapies, and even potential mutational profiles of their tumors [15,110]. There are also several trials that have yet to publish their final results. It is important to note, however, that caution must be exercised when translating clinical trial outcomes into real-world impact, as RCTs have strict eligibility criteria that may not reflect the actual population [91]. Furthermore, despite major scientific advancements, the survival of mRCC patients on second-line therapy remains limited, highlighting the need for further innovation and therapies. As a result, to all patients, we emphasize the importance of clinical trial participation and evaluation at high-volume cancer centers, such as those with National Cancer Institute designations. We also suggest actionable next steps to further propel the field including prioritizing research on combination therapies, optimizing trial designs, and investigating the effects of treatments based on specific patient characteristics such as disease biomarkers and metastatic location. Finally, we support the notion that special considerations should be made in the design of clinical trials to adequately study populations that are traditionally understudied in trials including patients with lower performance status and CNS/liver/bone metastasis.

## Figures and Tables

**Table 1.** Major clinical trials of second-line tyrosine kinase inhibitors.

Tyrosine Kinase Inhibitor	Clinical Trial(s)	Publication Year	Experimental Arm	Comparison Arm	Key Outcomes
Cabozantinib	METEOR [18,19]	July 2016	Cabozantinib	Everolimus	Median OS: HR 0.66 (95% CI 0.53-0.83, p=0.00026) PFS: HR 0.51 (0.41-0.62, p<0.0001) OR: cabozantinib 17% (13-22) vs. everolimus 3% (2-6) (p<0.0001) Second-line cabozantinib significantly improved patient survival compared to everolimus
	CaboPoint [5,22]	September 2024	Second-Line Cabozantinib after Nivolumab and Ipilimumab (Cohort A)	Second-Line Cabozantinib After Checkpoint Inhibitor and VEGF-Targeted Therapy (Cohort B)	ORR: cohort A 40.5% (95% 29.6-52.1) vs. cohort B 27.5% DCR: cohort A 84.8 months (75.0-91.9) vs. cohort B 80.0 (64.4-90.9) Median PFS: cohort A 10.9 months (8.2-14.2) vs. cohort B 8.3 (5.6-11.1) Cabozantinib effective in mRCC after CPI combination therapy
	BREAKPOINT [25]	February 2023	Cabozantinib after First-Line anti-PD-1/PD-L 1-Based Treatments	Phase II - None	mPFS: 8.3 months (95% CI 3.9-17.4) mOS: 13.8 months (7.7-29.0) ORR: 37.9% Even after ICIs, cabozantinib is active and has a manageable safety profile
Sorafenib	TARGET [30]	January 2007	Sorafenib	Placebo	PFS: sorafenib 5.5 months vs. placebo 2.8 months (HR 0.44, 95% CI 0.35-0.55, p<0.01) OS: HR 0.72 (95% CI 0.54-0.94, p=0.02) Compared to placebo, second-line sorafenib increased PFS
Axitinib	AXIS [31]	December 2011	Axitinib	Sorafenib	mPFS: axitinib 6.7 months vs. sorafenib 4.7 months (HR 0.665, 95% CI 0.544-0.812, p<0.0001) Compared to sorafenib, second-line axitinib significantly increased PFS
Tivozanib	TIVO-3 [32]	January 2020	Tivozanib	Sorafenib	mPFS: tivozanib 5.6 months (5.29-7.33) vs. sorafenib 3.9 months (3.71-5.55) (HR 0.73, 95% CI 0.56-0.94, p=0.016) Serious treatment-related adverse effects: tivozanib 11% vs. sorafenib 10% Compared with sorafenib, third or fourth-line tivozanib increased PFS and was better tolerated
Sunitinib	INMUNOSUN [35]	April 2022	Sunitinib	Phase II - None	OR: 19% (95% CI 2.3-35.8) mPFS: 5.6 months (95% CI 3.1-8.0 months) mOS: 23.5 months (95% CI 6.3-40.7) Stable response with clinical benefit: 86.7% (95% CI 70.7-100%) Sunitinib is active and can be safely used as a second-line option after ICI treatment
Lenvatinib	Motzer *et al.* [39]	November 2015	Lenvatinib (L) + Lenvatinib/Everolimus (LE)	Everolimus (E)	mPFS: - E 5.5 months (3.5-7.1) - LE 14.6 (5.9-20.1) (HR 0.40, 95% CI 0.24-0.68, p=0.0005) - L 7.4 (5.6-10.2) (HR 0.61, 0.38-0.98, p=0.048)

**Table 2.** Major clinical trials of second-line immune checkpoint inhibitors.

Immune Checkpoint Inhibitors	Clinical Trial(s)	Publication Year	Experimental Arm	Comparison Arm	Key Outcomes
Pembrolizumab	Lee *et al.* [55]	July 2021	Pembrolizumab/Lenvatinib Combination	Phase 1b/2 - None	ORR in ICI-Pretreated: 55.8% (95% CI 45.7-65.5) 66% had grade 3 or higher AEs Second-line pembrolizumab/lenvatinib demonstrated viability and had manageable safety profile
Atezolizumab	IMmotion150 [59]	May 2021	Second-Line Atezolizumab/bevacizumab after First-Line Atezolizumab or Sunitinib	Phase II - None	ORR: 27% (19-37) mPFS: 8.7 months (5.6-13.7) 83% had treatment-related adverse effects with 30% having grade 3/4 Second-line atezolizumab/bevacizumab combination therapy showed anti-tumorigenic activity and had a manageable side effect profile
	CONTACT-03 [60]	July 2023	Atezolizumab/Cabozantinib (AC)	Cabozantinib Monotherapy (C)	mPFS: AC 10.6 months (9.8-12.3) vs. C 10.8 (10.0-12.5) (HR 1.03, 95% CI 0.83-1.28, p=0.78) mOS: AC 25.7 months (21.5-not evaluable) vs. C not evaluable (21.1-not evaluable) (HR 0.94, 95% CI 0.70-1.27, p=0.69) Serious AEs: AC 48% vs. C 33% Adding atezolizumab to cabozantinib did not change survival but had increased toxicity
Nivolumab	CheckMate 025 [63]	November 2015	Nivolumab	Everolimus	mOS: nivolumab 25.0 months (95% CI 21.8-not estimable) vs. everolimus 19.6 (17.6-23.1) (HR 0.73, 0.57-0.93, p=0.002) ORR: nivolumab 25% vs. everolimus 5% (OR 5.98, 3.68-9.72, p<0.001) mPFS: nivolumab 4.6 months (3.7-5.4) vs. everolimus 4.4 (3.7-5.5) (HR 0.88, 0.75-1.03, p=0.11) Grade 3 or 4 TRAEs: nivolumab 19% vs. everolimus 37% Compared to everolimus, nivolumab had longer survival and fewer grade 3 or 4 AEs
	TiNivo-2 [64]	October 2024	Tivozanib/Nivolumab (TN)	Tivozanib (T)	mPFS: TN 5.7 months (4.0-7.4) vs. T 7.4 (5.6-9.2) (HR 1.10, 0.84-1.43, p=0.49) 1L ICI mPFS: TN 7.4 (5.6-9.6) vs. T 9.2 (7.4-10.0) 1L non-ICI mPFS: TN 3.7 (2.7-5.4) vs. T 3.7 (1.9-7.2) Serious AEs: TN 32% vs. T 37% mRCC patients should not consider ICI rechallenge but tivozanib monotherapy had a higher survival

**Table 3.** Ongoing major clinical trials of second-line therapies.

Ongoing Clinical Trial	Patient Accrual	Experimental Arm	Comparison Arm	Preliminary Outcome
CONTACT-03 [60]	07/28/2020-12/27/2021	Atezolizumab + Cabozantinib (AC)	Cabozantinib Monotherapy (C)	Adding atezolizumab to cabozantinib did not change survival but had increased toxicity
LITESPARK-011 [77]	02/25/2021-02/11/2026	Belzutifan + Lenvatinib	Cabozantinib	N/A
KEYMAKER-U03 [78]	12/17/2020-09/07/2025	Belzutifan + Lenvatinib	Phase I/II	ORR: 50% (95% CI 29-71) CBR: 54% (95% CI 33-74) Median PFS: 11.2 months (95% CI 4-NR) TRAEs in 93% Grade 3-4 TRAEs in 50%
LITESPARK-012 [102]	04/14/2021-10/29/2026	First-line pembrolizumab + belzutifan + lenvatinib	First-line pembrolizumab/lenvatinib	N/A
Choueiri *et al.* [80]	09/27/2018-07/14/2020	Belzutifan + cabozantinib	Phase II - None	OR: 30.8% (95% CI 18.7-45.1) Serious TRAEs in 29% Belzutifan/cabozantinib has demonstrated encouraging anti-tumorigenic activity
